# How to become immortal: let MEFs count the ways

**DOI:** 10.18632/aging.100129

**Published:** 2010-03-18

**Authors:** Adam Odell, Jon Askham, Catherine Whibley, Monica Hollstein

**Affiliations:** Faculty of Medicine and Health, University of Leeds, LIGHT Laboratories, Leeds LS2 9JT UK

**Keywords:** murine embryonic fibroblasts (MEFs); senescence; p53; p19/ARF; Hupki

## Abstract

Understanding the molecular mechanisms and biological consequences of genetic changes occurring during bypass of cellular senescence spans a broad area of medical research from the cancer field to regenerative medicine. Senescence escape and immortalisation have been intensively studied in murine embryonic fibroblasts as a model system, and are known to occur when the p53/ARF tumour suppressor pathway is disrupted. We showed recently that murine fibroblasts with a humanised p53 gene (Hupki cells, from a human p53 knock-in mouse model) first senesce, and then become immortalised in the same way as their homologues with normal murine p53. In both cell types, immortalised cultures frequently sustain either a p53 gene mutation matching a human tumour mutation and resulting in loss of p53 transcriptional transactivation, or a biallelic deletion at the p19/ARF locus.  Whilst these genetic events were not unexpected, we were surprised to find that a significant proportion of immortalised cell cultures apparently had neither a p53 mutation nor loss of p19/ARF.  Here we consider various routes to p53/ARF disruption in senescence bypass, and dysfunction of other tumour suppressor networks that may contribute to release from tenacious cell cycle arrest in senescent cultures.

## Culture shock causes rapid replicative senescence in primary MEFs with functional p53, but p53 deficient cells are resistant

Cellular senescence is an important defence mechanism against tumour metastasis, growth and progression [1,2]. Furthermore, in both humans and in mouse models, cancers can respond to chemotherapy by a massive senescence response followed by tumour cell clearance [3-5]. MEFs are a classic model system for studying cell senescence and immortalisation, with clear parallels to key genetic alterations during human tumourigenesis whilst offering expedient advantages over human cell cultures when exploring basic molecular mechanisms of senescence control and senescence bypass [6,7].  When explanted in vitro MEFs initially continue to replicate, but then rapidly undergo stress-associated senescence due to in vitro conditions, especially oxidative stress elicited by standard culturing conditions, which supply supraphysiological levels of oxygen [8].  DNA damage from reactive oxygen species is clearly a key factor in this senescence response [8,9]. As is well known from research in several laboratories, cells that sustain spontaneous damage to the p53/p19ARF pathway (p14 in humans) can overcome this replication block, leading to gradual outgrowth of an immortalised cell population with unlimited growth potential [10-14].

Our MEF cell line library comprises immortalised cell lines from cultures of fibroblasts derived from (a) embryos of a standard laboratory wild-type mouse strain (129/Sv), and (b) embryos from human p53 knock-in (Hupki) mice, which we constructed for our research on p53 biology [15-17].  The original Hupki mouse strain [15] harbours normal human p53 gene sequences encoding the DNA binding domain and the polyproline domain embedded in the endogenous murine p53 locus. This strain is phenotypically normal, not tumour-prone, and displays classical p53 wild-type responses, including DNA damage induced apoptosis, transcriptional transactivation of p53 target genes and stress-induced cellular senescence.  Curiously, a mouse strain in which the entire p53 sequence was replaced by the human counterpart lost wild-type p53 function due to abnormal interactions with the p53 negative regulator Mdm2 [18]. The Hupki strain is not p53-deficient, and can be used as a source of primary MEFs, thus allowing the extensive literature on MEF senescence bypass, and the database of human tumour suppressor mutations to be linked to specific mutations that support senescence bypass.  Using this approach, we have shown that the basic features of stress-induced senescence and immortalisation are comparable in MEFs from standard strain wild-type (WT) mice and Hupki mice. Studies from our laboratory with several hundred immortalised Hupki MEF cell lines have shown that the genetic alterations in p53 that lead to senescence bypass of MEFs are typical of human tumours. Missense point mutations in p53 and p19/ARF silencing by biallelic deletion, also common in human tumours,  are the two most common routes to spontaneous p53/p19ARF pathway inactivation in immortalised MEFs identified thus far ([16,19,20]  and unpublished observations).

**Figure 1.**
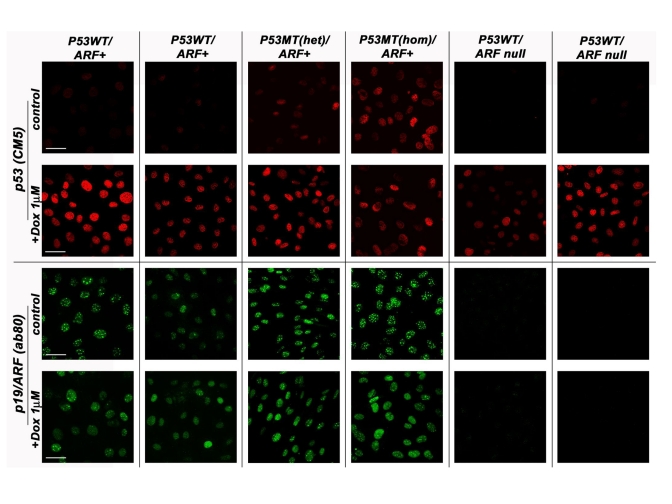
The p53/p19ARF status of various MEF lines derived from normal strain 129 mice. MEF cell lines genotyped as either wild-type (WT) p53 or mutant (MT) p53 (both heterozygous and homozygous) were compared against those carrying the p19/ARF deletion in their response to doxorubicin (8h, 1μM) treatment. Cells were methanol fixed and processed by indirect immunofluorescence confocal microscopy with either the anti-p53 CM5 (Novacastra) or anti-p19/ARF ab80 (AbCam) antibody. Scale bar represents 50μm. All samples were processed at the same intensity and magnification.

Whilst the prevalence of cell lines immortalised by p19/ARF biallelic deletion or p53 mutation was not unexpected (up to 50 % of cell lines, depending on immortalisation protocols), we were surprised to find that a significant fraction of cell lines (derived both from WT and Hupki primary cells) appeared to have retained WT p53 and p19/ARF expression, as examined by DNA sequencing or PCR amplification and promoter methylation analyses,  respectively ([20] and unpub-lished).  Probing by immunofluorochemistry for p53 nuclear accumulation and induction of p21/WAF1 following exposure of these cell lines to the DNA damaging agent doxorubicin (Figure 1), as well as detection of p19/ARF protein by immunoblotting [20] support the p53, p19/ARF wild-type status of these cell lines.  What might then be the genetic alterations that allowed these cells to bypass senescence? One possibility concerns the phenomenon of tumour suppressor haploinsufficiency in promoting cell growth (see insightful review by Quon and Berns, 2001) [21,22].    Contrary to the original 2-hit paradigm for tumour suppressor genes, where both alleles must be inactivated to elicit a growth promoting phenotype, ample evidence from in vivo and in vitro studies and human tumour analyses demonstrates that not only the absence, but also the moderate reduction of tumour suppressor gene products can be sufficient to alter growth phenotype. In the case of MEFs, as expected, cells from p53 null mice fail to senesce when explanted in vitro under standard culture conditions, but cells from heterozygous Hupki mice (progeny of Hupki and p53 null mice) also continue to grow when explanted, with only a brief slowing of doubling time after the first several passages (unpublished observations).  Some of these immortal cultures eventually do reveal loss of the WT allele with continued passaging, but others retain the original unmutated p53 allele. This suggests that the presence of only one WT allele in primary explanted MEFs may be sufficient to bypass senescence initially.  Conceivably, as passage number increases, so will the chances that the WT allele is eventually discarded, possibly provoking a jump in growth rate, as we have noted for some slow-growing MEF cell lines.

Some of the MEF cell lines from (homozygous) WT or Hupki mice that we have examined and tentatively classified as p53 and p19/ARF wild-type thus may in fact have only one normal allele of these suppressor genes, having suffered loss of one copy in vitro allowing unlimited growth in culture.

## Beyond p53/p19ARF

Given that at least half of the spontaneously arising >100 MEF cell lines we examined for p53 and p19/ARF aberrations appeared to have overcome senescence block upon explanting in vitro by mechanisms other than the 2 canonical genetic events (p53 mutation; p19 biallelic deletion), there could well be various alternative pathways to immortalisation not directly involving damage to the immediate p53/p19ARF axis.

Considerable effort is underway to identify the key regulators of senescence and immortalisation. Since the process of senescence bypass provides an intrinsic phenotypic readout of functionality and automatically generates cell lines amenable to subsequent analysis, reverse genetics is a powerful approach to deciphering the important molecular events involved in senescence control [23]. Several large-scale screens for genes involved in senescence/senescence bypass have been performed, including both gain-of-function screens involving the ectopic expression of cDNA libraries as well as loss-of-function screens involving the expression of antisense cDNA libraries and shRNA libraries. These screens have been performed in human and mouse cell models of both replicative and oncogene-induced senescence and each approach has identified different genes involved in senescence, providing novel and sometimes unexpected insights into the process (Table 1). Reassuringly, well established players in the master regulatory pathways of senescence (for example p53) have also been identified in these screens [24,25]. Indeed, inactivation of p53 is often used as a positive control in such experiments [24,26]. While p53 knock-down in senescent MEFs has been shown to reverse senescence [27], this may or may not be true of other genes involved in senescence which may require inactivation or expression prior to the acquisition of senescence.

The role of p53 mutation/p19ARF deletion in senescence bypass in MEFs reflects the importance of the p53/p19ARF axis as a master regulatory pathway of senescence in these cells.  What is apparent from genetic screening, as well as from other complementary work, is that many of the novel senescence-associated genes identified can also impact on this key pathway, both upstream and downstream of p53 [24,26,28-31].  The p16/pRb pathway, another senescence master regulator, is also commonly affected by novel senescence-associated molecules [28,32].  A number of additional interacting signaling pathways have been implicated in the induction or bypass of senescence including the RAS/MAPK pathway [25,33,34], the AKT pathway [35-37] and the JNK pathway [38,39], although the relative contribution of these to the senescent phenotype appears to be dependent on species, cell type and the pro-senescence stimulus.

## Concluding remarks: Senescence - good or bad?

An entirely new aspect to the importance of cellular senescence has recently surfaced from experiments to produce iPS (induced pluripotent stem) cells from embryonic fibroblasts.  It has been shown that senescence provides a progressive barrier to conversion of primary MEFs (and indeed other types of differentiated cells) to pluripotency.  Crucially, disruption of the p53/p19ARF signalling axis greatly increased efficiency of their conversion [40,41].  Furthermore, genetic ablation of p53 in cells normally considered refractory to reprogramming into pluripotent stem cells can overcome this block [40].  Clearly, senescence is a key process to target in optimising strategies to enhance somatic cell reprogramming.  Identification of factors influencing senescence will reveal novel genes/pathways to modulate that could enhance conversion to pluripotency without compromising  genetic  integrity, expediting potential applications for iPS cells in regenerative medicine.

**Table 1.** Novel genes identified in reverse-genetics senescence bypass screens. The table shows the diversity of genes which either promote senescence or its bypass as identified in cellular screens for senescence bypass. Genes well known to be important in cellular senescence such as p53, p21 and PAI-1 are not included here. ^a^Other genes identified in this screen: BNIP3L, BIN1, HSPA9, IL1R1, PEA15, RAP1GAP, DMTF1, FOXA1, IRF1, MEN1, HIRA, SMARCB1, FBXO31, NF2 [25]. ^b^Additional genes identified in this screen: RPS6KA6, HTATIP, HDAC4, SAH3, CCT2 [24].

**Gene**	**Promotion or inhibition of senescence**	**Potential senescence-associated pathway/mechanism of action**	**Biological Function**	**Refe-rence**	**Cell type**
**BCL6**	inhibition	Induces cyclin D1 expression and renders cells unresponsive to antiproliferative signals from the p19(ARF)-p53 pathway	Transcription factor	[29]	MEFs, human B cells
**Bub1**	inhibition	Bub1 RNAi induces senescence. Bub1 expression does not extend lifespan	Mitotic checkpoint Ser/Thr kinase	[25^a^, 28, 42]	Primary MEFs
**Csn2**	promotion	Inactivation inhibits p53 transcriptional activity and confers resistance to both p53- and p16INK4a-induced proliferation arrest	Component of the Cop9 signalosome	[28]	Primary MEFs
**Brf1**	promotion	Inhibition of p53 transcription and reduction p16ink4a-induced arrest	Subunit of the RNA polymerase II complex	[28]	Primary MEFs
**Aldose Reductase**	promotion	Inhibition of p53 transcription and reduction p16ink4a-induced arrest	Metabolic enzyme - glucose metabolism	[28]	Primary MEFs
**Tid1**	induction	Tid1 is a repressor of NF-κB signaling	DNA-J like protein which functions as a co-chaperone	[43]	Rat embryo fibroblasts
**hDRIL1**	inhibition	Renders primary MEFs unresponsive to RAS(V12)-induced anti-proliferative signaling by p19(ARF)/p53/p21(CIP1), as well as by p16(INK4a) Binds E2F1 and induces Cyclin E1	Transcription factor	[32]	MEFs RAS^V12^ induced senescence
**CBX7**	inhibition	Controls cellular lifespan through regulation of both the p16(Ink4a)/Rb and the Arf/p53 pathways Represses INK4a-ARF locus	Transcription factor	[44]	Normal human prostate epithelial cells
**LPA(2)**	inhibition	E2F induction	Phospholipid receptor	[45]	Mouse neuronal cells
**Dbs**	inhibition	E2F induction	Rho-specific guanine nucleotide exchange factor	[45]	Mouse neuronal cells
**TBX2**	inhibition	TBX2 represses the Cdkn2a (p19(ARF)) promoter	Transcription factor	[31]	*Bmi1*^-/-^ MEFs
**TBX3**	inhibition	TBX-3 potently represses expression of both mouse p19(ARF) and human p14(ARF)	Transcription factor	[30]	Mouse neuronal cells
**Topo1**	promotion	DNA damage-ATM-p53	Nuclear enzyme regulating DNA structure Relaxes positively and negatively supercoiled DNA	[26]	Normal human cells
**IGFBP7**	promotion	MEK, ERK pathway In Brafv600E-mediated senescence, IGFBP7 inhibits BRAF-MEK-ERK signaling by inducing RKIP, which prevents BRAF from phosphorylating MEK	Ser/Thr protein kinase, oncogene Growth factor receptor	[25, 46]	Human primary fibroblasts, melanocytes
**KLF4**	promotion	p53 pathway Suppresses the expression of p53 by directly acting on its promoter Induces p21	Transcription factor	[47]	Conditionally immortalized MEFs co-expressing RAS^V12^
**SAHH**	promotion	p53 pathway SAHH inactivation inhibits p53 transcriptional activity		[24^b^, 28, 48]	Primary human fibroblasts, Primary MEFs
**CXCR2 (IL8RB)**	promotion	p53 pathway CXCR2 knock-down alleviates both replicative and oncogene-induced senescence and diminishes the DNA-damage response.	Chemokine receptor	[49]	Primary human fibroblasts
